# Fourier Transform Infrared Spectroscopic Imaging of Fracture Healing in the Normal Mouse

**DOI:** 10.1155/2015/659473

**Published:** 2015-01-01

**Authors:** Hans Gollwitzer, Xu Yang, Lyudmila Spevak, Lyudmila Lukashova, Allina Nocon, Kara Fields, Nancy Pleshko, Hayden William Courtland, Mathias P. Bostrom, Adele L. Boskey

**Affiliations:** 1 Hospital for Special Surgery, 535 E. 70th Street, New York, NY 10021, USA; 2 Klinik für Orthopädie und Sportorthopädie, Technische Universität München, Ismaningerstrasse 22, 81675 Munich, Germany; 3 Temple University College of Engineering, 1947 N. 12th Street, Philadelphia, PA 19122, USA

## Abstract

Fourier transform infrared spectroscopic imaging (FTIRI) was used to study bone healing with spatial analysis of various callus tissues in wild type mice. Femoral fractures were produced in 28 male C57BL mice by osteotomy. Animals were sacrificed at 1, 2, 4, and 8 weeks to obtain callus tissue at well-defined healing stages. Following microcomputerized tomography, bone samples were cut in consecutive sections for FTIRI and histology, allowing for spatial correlation of both imaging methods in different callus areas (early calcified cartilage, woven bone, areas of intramembranous and endochondral bone formation). Based on FTIRI, mineral/matrix ratio increased significantly during the first 4 weeks of fracture healing in all callus areas and correlated with bone mineral density measured by micro-CT. Carbonate/phosphate ratio was elevated in newly formed calcified tissue and at week 2 attained values comparable to cortical bone. Collagen maturity and mineral crystallinity increased during weeks 1–8 in most tissues while acid phosphate substitution decreased. Temporal and callus area dependent changes were detected throughout the healing period. These data assert the usefulness of FTIRI for evaluation of fracture healing in the mouse and its potential to evaluate pathologic fracture healing and the effects of therapeutic interventions.

## 1. Introduction

Fractures and nonunions of normal and osteoporotic bone are of great clinical relevance, and a wealth of physical and pharmacological methods are currently being evaluated to improve the healing response of fractured bone [1]. Commonly used drugs such as bisphosphonates and parathyroid hormone are known to influence callus size and strength, callus mineralization, and callus remodeling [2, 3] and the possibility exists that other pharmacologic agents, such as beta-blockers and other anabolic agents, might improve fracture healing as well [1, 4, 5]. These approaches require valid preclinical monitoring of beneficial and deleterious influences of these drugs on fracture healing and bone strength. It is our hypothesis that Fourier transform infrared imaging spectroscopy (FTIRI) concomitant with histologic evaluation can better characterize the events of fracture healing in the presence or absence of pharmaceutical treatments.

Fracture healing is a complex process that mimics endochondral ossification, the process by which bones grow in length during development. The spatial and temporal distribution of the different stages of development within the callus, are more heterogeneous than those in the epiphyseal growth plate [5, 6]. FTIRI, in which an infrared spectrometer is coupled to a light microscope, provides a chemical photograph of the composition of thin sections of tissue [7].

The sequence of fracture healing corresponds to the classic four-stage model (inflammatory response, soft callus formation, hard callus formation, initial bony union and bone remodeling (Table 1)) and to the time periods investigated by FTIRI in this study. Although this model describes the fundamental events that occur during the healing of a fracture, there are significant overlaps between the stages [5]. For example, the replacement of peripheral callus with lamellar bone remodeling due to intramembranous bone formation (Stage 4) occurs concurrently with endochondral cartilage mineralization (Stage 2), vascular invasion, and woven bone formation (Stage 3). During fracture healing, a cartilage anlagen consisting predominately of type II collagen is replaced primarily by type I collagen. Mineralization of this new matrix gives rise to bone [5, 6]. It is important to note that while the four-stage model is applicable to fracture areas with endochondral bone formation, areas of intramembranous repair also exist in fracture callus and directly result in hard callus formation from periosteal cells without transformation of cartilage, and the four-stage model is a non-sequitor. This emphasizes the need to separately investigate the various areas of callus formation during fracture repair prior to and after the administration of drugs.

The volume and density of deposited mineral, bone geometry, and bone architecture are generally studied as predictors of bone strength and fracture risk [4, 8]. These parameters, however, do not sufficiently predict whole bone mechanical properties, and the additional evaluation of intrinsic factors such as bone mineral density distribution, architectural anisotropy, mineral composition, crystallite size and perfection, and crystallite orientation has been shown as valuable predictors of fragility fractures [4, 9, 10]. Sensitive diagnostic tools are therefore needed to monitor the influences of therapeutics and bone pathologies on the complex interactions during fracture healing. FTIRI provides a tool for monitoring the distribution and composition of materials within the callus and provides a unique opportunity for the chemical characterization of nondemineralized tissue sections at the ultrastructural level with a spatial resolution of about 7 *μ*m [7, 10].

Both collagen deposition and matrix mineralization can be monitored spectroscopically. It has been previously shown that FTIRI provides parameters that can be used as sensitive indices of bone mineral content, mineral crystal size and perfection, acid phosphate content, and collagen maturity [7, 9, 10]. While FTIRI cannot differentiate types of collagen or recognize advanced glycation end products [11] formed on the collagen, it can distinguish changes in both mineral and matrix maturity. FTIRI has been used to study pharmacologic effects on normal and diseased bone in humans and in various normal and mutant animals [12–18], cartilage composition and degeneration [19–21] as well as collagen orientation in cartilage [22]. FTIRI and FTIR microspectroscopy were previously used to provide insights into bone mineralization processes in both fracture healing and in the epiphyseal growth plate [18, 23]. Ouyang et al. used FTIR microspectroscopy to describe the influence of estrogen on fracture healing in a rat model of osteoporosis [23]. A random area of fracture callus adjacent to the osteotomy site was investigated without benefit of histological differentiation. This may have given rise to conflicting results due to the presence of different tissues types within the callus. Similarly, a study of fracture healing in IL-6 knockout and wild-type mice used the center of the callus and adjacent cortical bone to demonstrate FTIRI compositional variation during the first four weeks of healing at the end of the pubertal growth phase [18]. The purpose of the present study is to characterize the compositional changes in histologically defined discrete regions of the callus during the course of fracture healing in the adult male mouse and to test the hypothesis that this evaluation can better characterize the events of fracture healing in the normal mouse.

## 2. Materials and Methods

### 2.1. Animal Model and Surgical Technique

All experimental procedures were in compliance with the principles in the “Care and Use of Animals” and were approved by the Hospital for Special Surgery’s Institutional Animal Care and Use Committee. Fracture healing was investigated in 28 male C57BL/6 wild-type mice (Jackson Laboratories, Bar Harbor, Maine). The starting age of the mice was at 14 weeks. This age represents the later growth phase in the mouse, just prior to the age at which peak bone mass is obtained [24, 25]. Transverse mid-diaphyseal fractures were produced by osteotomy as previously described [26], under general anesthesia, using 1.5mL (150mg) Ketamine HCL (Ketaset, Dodge Animal Health, Fort Dodge, IA), 1.5mL (30mg) Xylazine (Ben Venue Laboratories, Bedford, OH), and 0.5mL (5mg) Acepromazine Maleate Injection (Boehringer Ingelheim Vetmedica, Inc., St. Joseph, MO). The combined dose of anesthetic was 0.5 mL/kg (body weight of mouse).

Surgical procedure began with the ventral portion of the right femur and knee shaved and disinfected with iodine solution. An incision was made at the medial aspect of the knee and extended proximally to the midshaft of the femur. The joint capsule of the knee was opened just medial to the patella and the intercondylar notch was exposed. A 25-gauge needle was used to bore a hole in the distal femur and was introduced into the medullary canal serving as an intramedullary rod. Deep dissection was extended proximally and a transverse fracture was created at the femoral midshaft using a 0.3mm burr. The resulting bone fragments were left visibly aligned and the intramedullary needle was advanced well over the fracture site leaving the proximal and distal bone fragment in contact. Finally, the needle was cut to an appropriate length and the wound was irrigated and closed with sutures. Postoperatively, completion of the fracture and contact of the bone fragments was verified by X-ray. All mice received 0.05 ng/kg buprenorphine (Reckitt Benckiser Pharmaceuticals, Inc., Richmond, VA) subcutaneously *ad libitum* for pain relief, and full cage activity was allowed after the operation. Animals were sacrificed with carbon dioxide inhalation at 1 week (*n* = 6), 2 weeks (*n* = 8), 4 weeks (*n* = 8), and 8 weeks (*n* = 6) after the fracture, and femurs were collected and immediately fixed in ethanol (70% v/v).

### 2.2. Microcomputed Tomography

Mineralized tissue formation in the fracture callus was assessed prior to FTIRI analysis by quantitative microcomputed tomography (micro-CT). Femurs were placed in a tube filled with aqueous ethanol (70% v/v) which also included a phantom containing air, saline, and a bone analogue (1.18 g/cc) for mineral density calibration. Scans were carried out at 80V and 80 *μ*A with an MS-8 small animal scanner (GE Healthcare Technologies, Waukesha, WI). Correction, reconstruction, and quantitative analysis of the scans were accomplished with MicroView software (GE Healthcare Technologies, Waukesha, WI). For callus measurements, only newly formed callus areas surrounding the medullary canal and the cortex (external callus) were analyzed since medullary callus formation was influenced by the placement of the intramedullary rod. First, the outer boundaries of the entire callus were delineated with a best-fit volume of interest (VOI). Then, a second VOI was created by following the outer borders of the original cortex which was equivalent to the original bone without the external fracture callus. By subtracting the VOIs, the newly formed external fracture callus was defined. A third VOI was generated circumscribing cortical bone remote from the fracture site. A specimen-specific threshold was used to minimize any variability among the individual scans. Mineralized tissue in the images was thresholded using 25% of the mineral attenuation value of cortical bone for all specimens [29]. The following micro-CT parameters were calculated: total mineral content (TMC), bone volume fraction (BV/TV), callus volume, and volume of bone. Tissue mineral density (TMD), a measure of tissue mineralization independent of bone volume or amount, was calculated for both callus and cortex.

### 2.3. Histology

**Figure 1:**
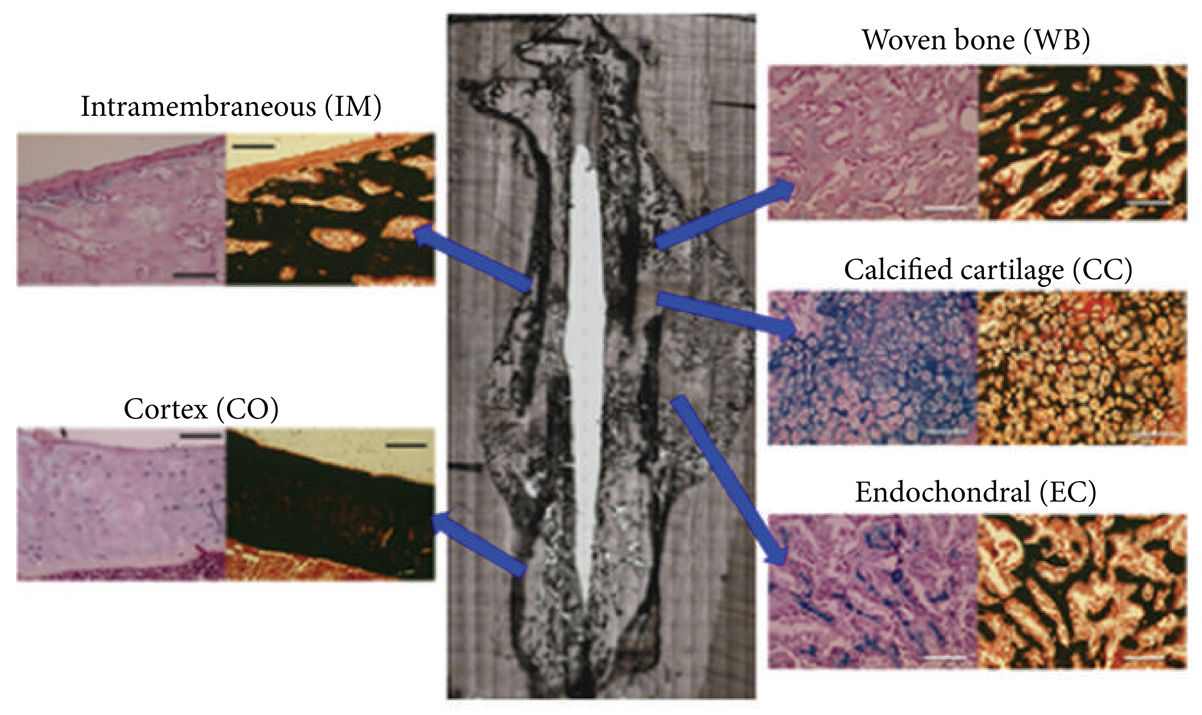
Different histological areas within the fracture callus that were studied by FTIRI. Normal cortical bone (CO) remote of the callus served as reference bone. Scale bars = 100 *μ*m. (CC) areas of calcified cartilage; (EC) = areas of endochondral new bone formation; (WB) areas of woven bone formation; (IM) = areas of intramembranous new bone formation. Correlation of TMD and mineral/matrix ratio in the fracture callus is in Figure 2(c). The mineral/matrix values for each area of the callus were plotted versus the time at which a given TMD was measured. The least square best line shown was fitted through all these data points.

Following micro-CT analysis, femora were embedded in PMMA and cut into adjacent consecutive longitudinal nondecalcified sections of 6 *μ*m (for histology) and 1–2 *μ*m (for FTIRI). Sections for histology were stained with Alcian blue and von Kossa stains to allow for differentiation of the various callus areas, specifically areas with proteoglycan content (Alcian blue stain) and those of mineralized tissue (von Kossa stain). Five histologic areas in the callus were chosen to represent different stages and types of fracture healing within each specimen (Figure 1) and included (1) early calcified cartilage with intact chondrocytes and interstitial mineral deposits (CC), (2) areas of endochondral bone formation with mainly mineralized tissue and positive staining for proteoglycans (EC), (3) woven bone without signs of cartilage or proteoglycan remainders (WB), (4) areas of intramembranous bone formation (IM), and (5) cortical bone remote from the fracture site (CO).

### 2.4. Fourier Transform Infrared Imaging

FTIRI data was collected with a Spectrum Spotlight 300 FT-IRI system (Perkin Elmer Inc., Waltham, MA). Data was processed with ISYS 3.1 software (Malvern Industries, formerly Spectral Dimensions Inc., Olney, MD) as described elsewhere [7, 9, 10]. In brief, the 1–2 *μ*m thick sections were mounted on BaF_2_ windows. Areas without tissue sections served as background. Spectra were collected from 800 cm^−1^ to 2000 cm^−1^ at 8 cm^−1^ spectral resolution from tissue, background, and regions of only PMMA. Specific areas of the newly formed external callus were scanned that corresponded to the previously defined histological types of mineralized tissue: CC (calcified cartilage), EC (endochondral bone), WB (woven bone), IM (intramembranous bone), and CO (cortical bone). Following base-lining using a linear baseline, PMMA contributions were spectrally subtracted [9] and pixels with absorbance values less than zero were masked (ISys software). FTIRI scans were optically aligned with selected histological areas. A total of 6–8 images were acquired from each mouse femur at each time point. All scanned fields covered the maximum area of each tissue type in each section. CC (calcified cartilage) and EC (endochondral bone) were not present at all-time points, and thus were only evaluated at 1 and 2 weeks. Cortical bone remote from the callus served as a control tissue. Five FTIRI compositional parameters were calculated for each image: mineral content (mineral/matrix peak area ratio), mineral crystallinity (intensity ratio), mineral carbonate content (carbonate/phosphate peak area ratio), and acid phosphate substitution (intensity ratio). The wavenumber range used to define these parameters and references to the literature in which they were validated are summarized in Table 2. These parameters were verified from randomly selected spectra in the different callus areas from which second-derivatives were displayed.

### 2.5. Statistical Analysis

Continuous variables were presented as means with standard deviations and medians with 1st and 3rd quartiles. The generalized estimating equations (GEE) approach with compound symmetric correlation structure was used to assess the association between time (1, 2, 4, and 8 weeks) and tissue type (CC, CO, EC, IM, and WB) with all five FTIRI measurements: (1) mineral matrix ratio, (2) carbonate mineral ratio, (3) HA crystallinity, (4) acid phosphate, and (5) collagen crosslinks. The GEE approach was used to account for the presence of data from multiple tissues from the same animal. Although there was no evidence of an overall interaction between time and tissue with respect to any of the FTIRI outcomes, the interaction terms were retained in the models for exploratory analyses. These exploratory analyses included comparison of mean FTIRI value between tissues while holding time point constant and comparison of mean FTIRI value between time points while holding tissue constant.

One-way analysis of variance (ANOVA) was used to assess the association between time (1, 2, 4, and 8 weeks) and each of the following micro-CT measurements: (1) TMC, (2) TMD, (3) TMD cortex, (4) BVF, (5) Volume, and (6) Volume of bone. When an ANOVA *P* value was <0.05, mean micro-CT values were compared in between pairs of time points using independent-samples *t*-tests.

All statistical analyses were performed with SAS Version 9.3 (SAS Institute, Cary, NC). All statistical tests were two sided with a *P* value < 0.05 considered statistically significant. *P* values were adjusted for multiple comparisons using the Tukey-Kramer method.

## 3. Results

### 3.1. Micro-CT

**Figure 2:**
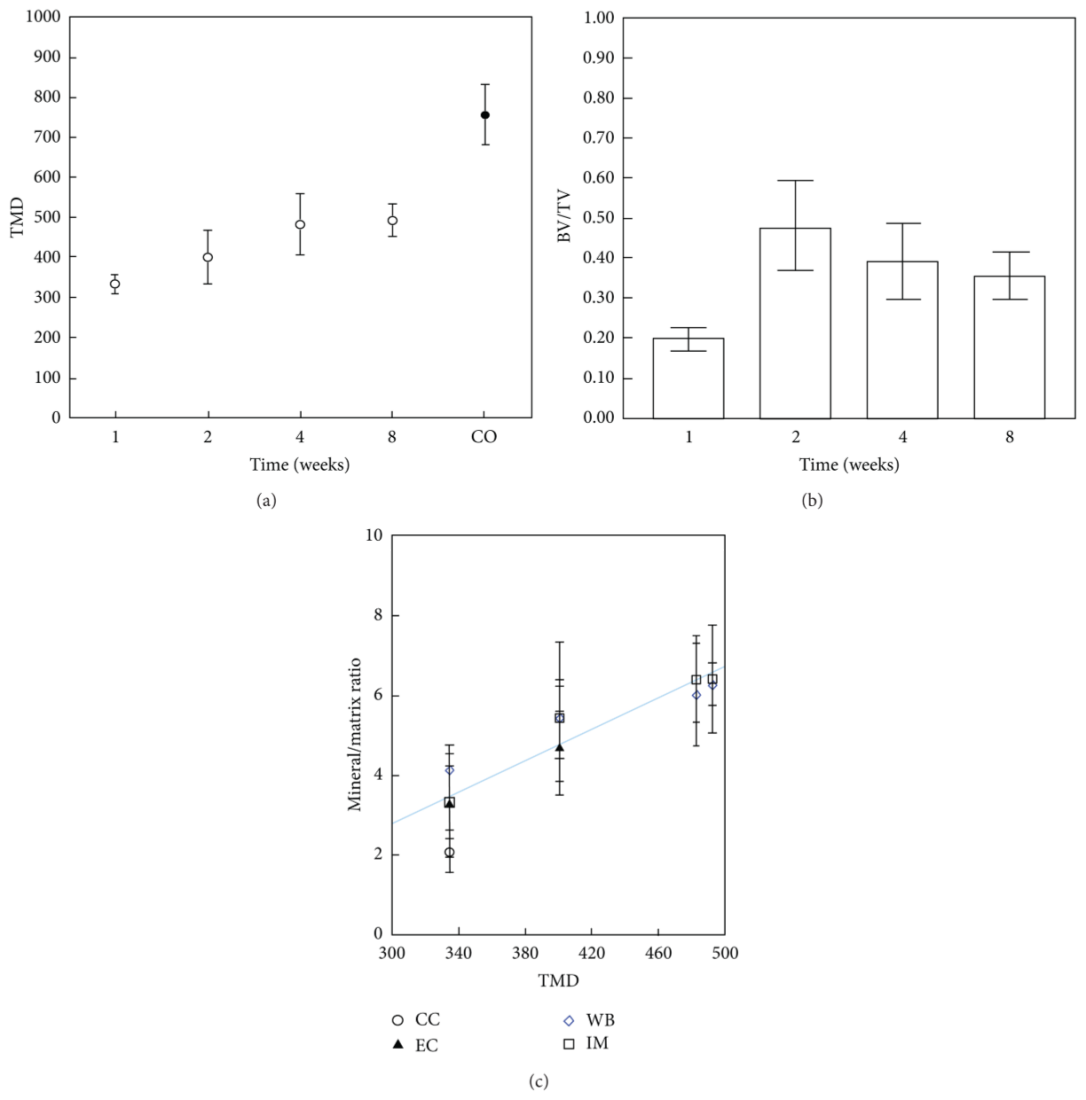
Microcomputed tomography shows changes in fracture callus with time. (a) Tissue mineral density (TMD; g/cc) increases linearly from weeks 1–4 but never reaches value of cortical bone. (b) Bone volume fraction (BV/TV) decreases from week 2–8. (c) Mineral/matrix ratio as determined by FTIRI in the different regions of the callus correlated with TMD measured at that same point in time.

Micro-CT analysis revealed an increase in callus volume and tissue mineral density (TMD) as a function healing time. TMD increased linearly up to 4 weeks (*P* < 0.001), did not increase significantly from 4–8 weeks, and did not reach the value of control cortical bone (CO) (Figure 2(a)). There were significant increases in bone volume fraction between weeks 1 and 2 and between weeks 1 and 4 (Figure 2(b)). Summary statistics for the micro-CT data are presented in Table 3.

### 3.2. FTIRI Data

**Figure 3:**
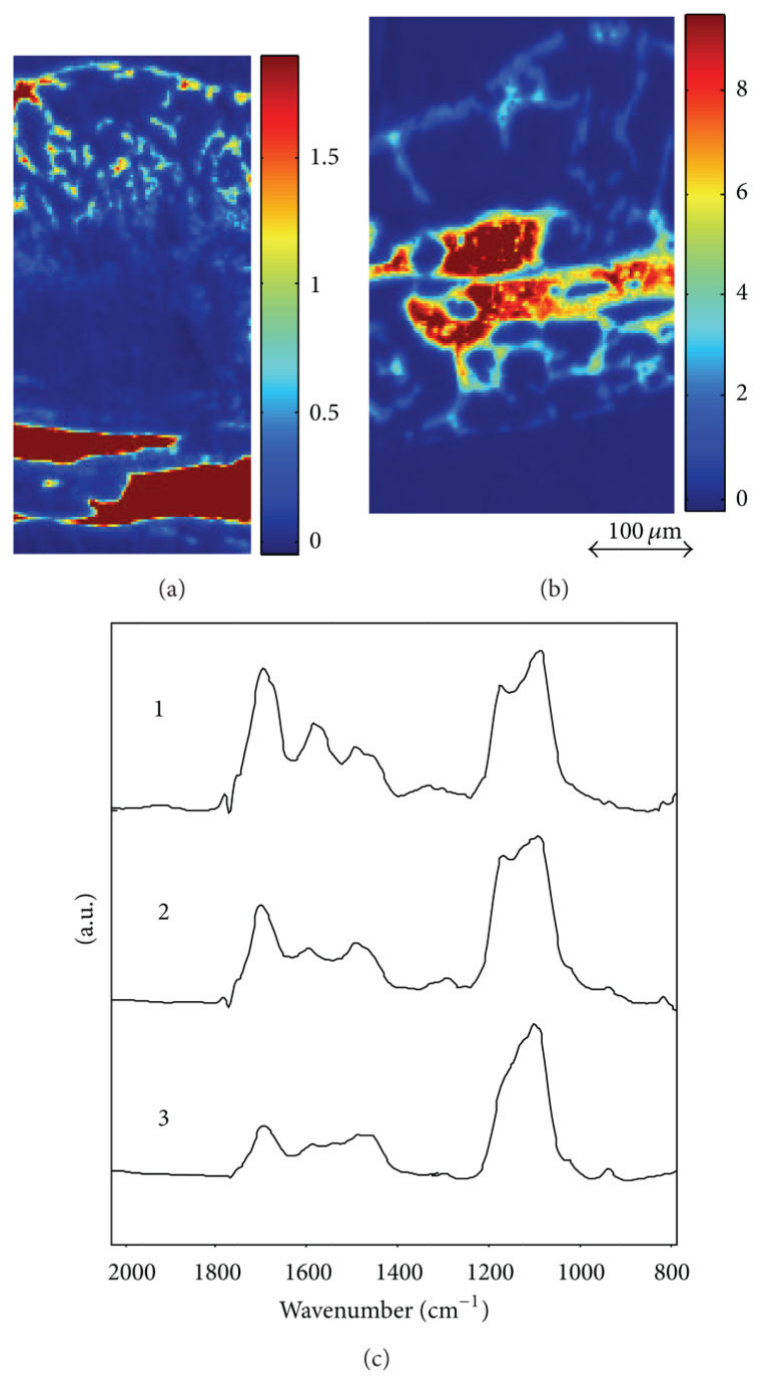
Mineral/matrix in whole callus and underlying fractured bone at 2 (a) and 4 (b) weeks after fracture. Figure (c) shows typical spectra from (1) callus, (2) woven bone, and (3) cortical bone. Note the strong subband at 1112 cm^−1^ in (1) and (2).

**Figure 4:**
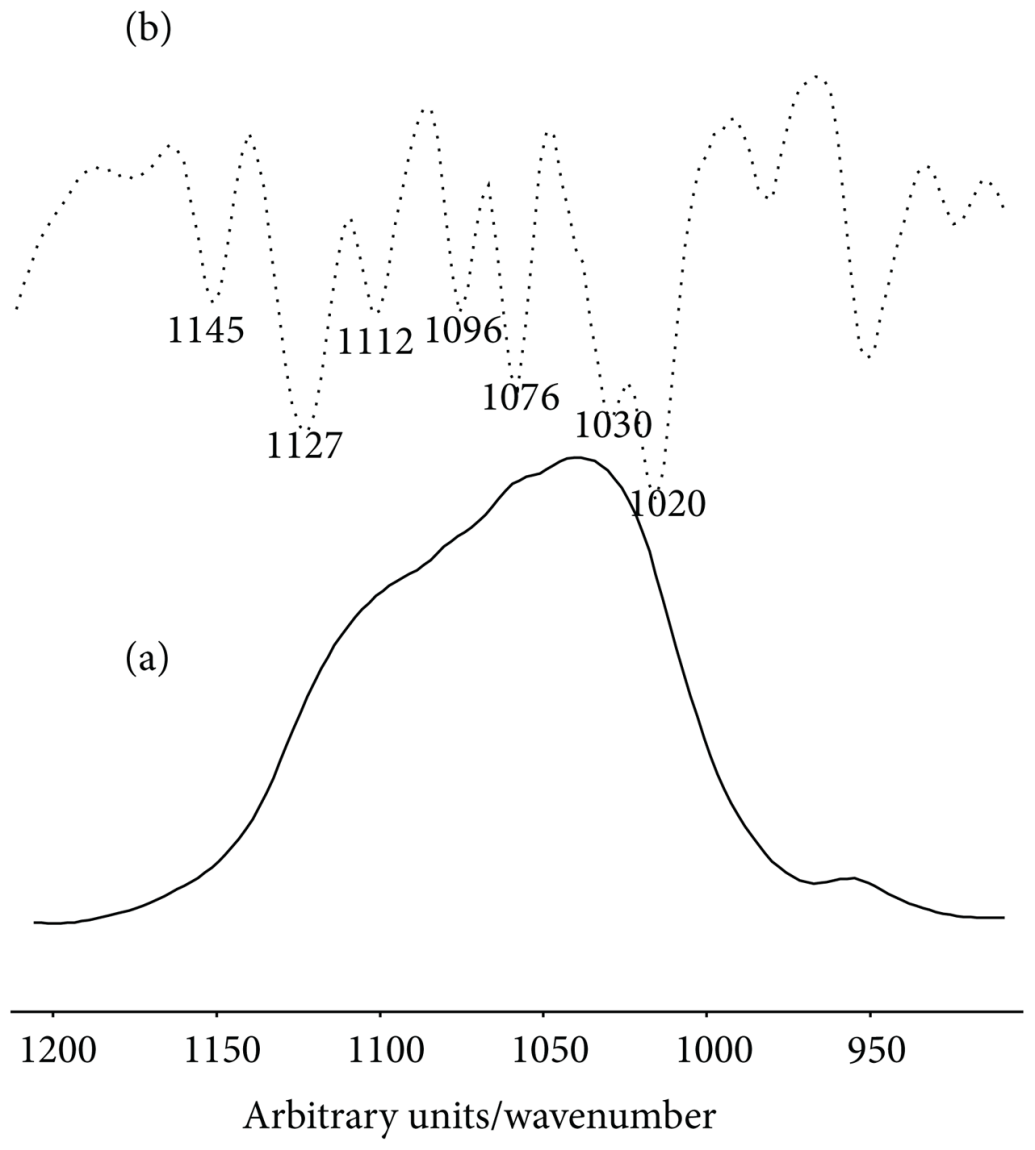
Second-derivative spectra of woven bone at 2 weeks of healing. Note the position of the peaks agree with the parameters used to characterize the mineral shown in Table 2.

**Figure 5:**
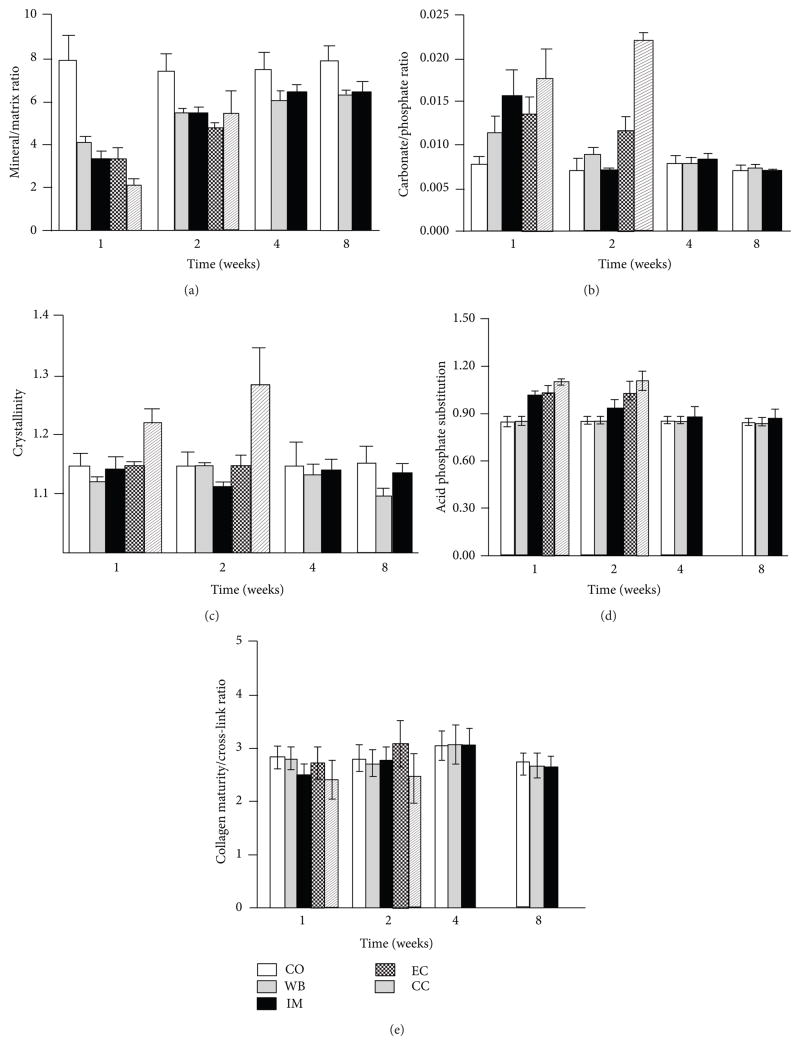
Quantitative results of FTIRI of the various callus tissues and cortical bone at different time-points. Each value represents the mean of 6 to 8 bone specimens of equivalent tissue type and time point (CC: *N* = 3). (a) Mineral/matrix ratio; (b) carbonate/phosphate ratio; (c) hydroxyapatite crystallinity; (d) acid phosphate substitution into hydroxyapatite. (e) Collagen maturity. CO = cortical bone; WB = woven bone; IM = area of intramembranous new bone formation; EC = area of endochondral new bone formation; CC = early calcified cartilage.

The regions chosen for FTIRI analyses in the fracture callus corresponded to histologically distinct areas (Figure 1). FTIR spectra of newly formed mineralized tissue (Figure 3(a)) in the callus and adjacent bone during the 8-week study period of fracture healing showed increases in the relative height of the mineral peak and changes in peak shape (Figure 3(b)). The images of the whole callus revealed a time dependent change in mineral/matrix ratio (Figure 3(a)). Second derivative spectra confirmed the peak positions (Figure 4) as illustrated here for woven bone at 2 weeks. The mean values obtained for these parameters in the various histologic regions as a function of time are summarized in Figure 5, a composite, with the statistical comparisons in Tables 4(a)–4(e). Mineral/matrix ratio increased continuously and significantly with healing time in all areas of the newly formed callus (Figure 5(a), Table 4(a)) until week 4 (*P* < 0.001) and correlated significantly with TMD as measured by micro-CT (Spearman’s rank correlation coefficient = 0.688, *P* < 0.01; Figure 2(c)). Mineral/matrix ratio was significantly higher in cortical bone compared to all callus areas (*P* < 0.025) at all time points; there were no significant differences among the mineral/matrix ratios when various calcified tissues in the callus were compared at corresponding time-points (*P* > 0.05). The mineral content in very early calcified cartilage appeared lower than the other callus areas, although the difference was not statistically significant (*P* > 0.05).

Carbonate/phosphate ratio (Figure 5(b), Table 4(b)) was significantly elevated in all callus areas at weeks 1 and 2 compared to both cortical bone and woven bone (*P* < 0.015). This increase may reflect the inclusion of nonmineralized tissue in the areas examined. At 2 weeks, the value of intramembranous bone carbonate/phosphate ratio was equivalent to that of cortical bone (*P* > 0.05). Thereafter, carbonate/phosphate ratio decreased rapidly and reached the values of cortical bone in all areas except woven bone which remained elevated at 8 weeks.

Mineral crystallinity (Figure 5(c), Table 4(c)), a parameter previously shown to be indicative of crystal size and perfection as measured by X-ray diffraction line broadening analysis [7], increased significantly in woven bone in the span of 1–2 weeks and decreased between weeks 2 and 8. At week 8, crystallinity in cortical bone was greater than that in woven bone and intramembranous bone. Contrastingly, acid phosphate substitution decreased significantly with healing time and callus maturation in intramembranous bone, woven bone, and calcified cartilage (Figure 5(d), Table 4(d)). Acid phosphate substitution in intramembranous bone areas was higher at one and two weeks than in the same tissue type at 4 and 8 weeks. At 4 weeks, cortical bone had lower acid phosphate substitution than intramembranous bone; they were equivalent by 8 weeks.

Collagen maturity/cross-link ratio increased between weeks 1 and 4 and weeks 4 and 8 in intramembranous bone. Collagen maturity was lower in calcified cartilage than woven bone and cortical bone at 1 week (Figure 5(e), Table 4(e)). There were no other significant changes in this ratio.

## 4. Discussion

This study demonstrated the ability of FTIRI to characterize mineral and matrix changes in distinct histological areas of the healing fracture callus, indicating the usefulness of these techniques for monitoring drug effects in similar models. The present study is the first comprehensive FTIRI assessment of fracture healing, with differential analysis of the various callus tissue types. Changes in mineral content (mineral/matrix ratio), composition (carbonate and acid phosphate substitution), and crystal size/perfection (crystallinity) along with collagen maturity (cross-links) were documented in each tissue type as a function of healing time.

FTIRI was previously used to describe the composition of rat fracture callus at a single point in time [30], demonstrating a correlation between FTIRI data and small angle scattering data in the same bone. FTIR microspectroscopy (FTIRM) described differences in whole callus composition in the rat as a function of ovariectomy, estrogen treatment, and time of healing [23]. FTIRI had been used by our group to study differences in mineralization processes during fracture healing in wild-type and IL-6 knock-out mice [18]. A significant limitation of these previous fracture studies was the investigation of a random callus area close to the fracture site, without histological differentiation. These results may have limited value due to the simultaneous investigation of both woven bone and calcified cartilage in the healing callus.

To address this prior limitation, we investigated callus areas by FTIRI in regions corresponding to specific histological tissue types at different stages of endochondral and intramembranous new bone formation. Since endochondral bone formation is a continuous process from early calcified cartilage to woven bone and the different stages cannot always be clearly discerned, we expected to find this continuous transition to be similarly reflected in the FTIRI results. Mineral/ matrix ratio within the callus in most tissue types, with the exception of cortical bone which was invariant, increased continuously for 4 weeks post-fracture and remained constant thereafter without reaching the mineral content of the very dense and highly-mineralized cortical bone. As expected, the mineral content in very early calcified tissues was the lowest, with significant differences among different tissue types at earlier time points (weeks 1 and 2). Demonstrated previously, mineral/matrix ratio corresponded linearly with ash weight [31]. We compared mineral/matrix ratio as measured by FTIRI with BMD in micro-CT, and found significant correlation for both metrics of measurement of mineral density in the fracture callus. Correlation of mineral/matrix ratio and TMD levels should be limited, since FTIRI assesses two-dimensional tissue sections whereas micro-CT provides three-dimensional analysis; however, as here, a correlation has been reported in other studies [32]. It is important to note that others have correlated FTIR microscopy findings with histology, for example, identifying crystallinity and type of carbonate substitution in surgically excised human calcific tendinitis and rotator cuff deposits [33, 34].

Carbonate substitution into hydroxyapatite in the callus components decreased rapidly in all newly formed bone areas. The elevated carbonate/phosphate ratio at week 1 may reflect the decreased mineral (phosphate) content in the tissue; however, since carbonate/phosphate ratio has been regarded as a measure of bone turnover [35], an increase in more actively remodeling sites in these values, these values may reflect the observed increased remodeling that is part of the fracture healing process. Carbonate/phosphate rapidly decreased in both the intramembranous and woven bone areas of the callus and reached values comparable to cortical bone by week 2. Elevated carbonate/phosphate was still detectable in cartilaginous callus tissues (calcified cartilage and endochondral bone) at week 2. Presence of cartilage, active mineralization, and remodeling, rapidly disappeared from week 4 onward. This is in agreement with a recent Raman spectroscopy study in a mouse model of osteogenesis imperfecta where the control limb values in the wild type mouse (treated and untreated models) had a lower carbonate/phosphate ratio than the genotype-and treatment-matched callus values [29]. The results are generally consistent with previous studies of fracture healing in the ovariectomized rat which heals more slowly [23] and where carbonate/phosphate ratio in the entire callus decreased continuously from 4 weeks to 12 weeks. Carbonate substitutes for both OH and PO_4_ in the hydroxyapatite lattice. There is also a labile carbonate that is not associated with these positions [36, 37]. Remodeling and age progression causes these substituents to reach a constant level as the labile carbonate is removed [38]. Contrary to the decrease in carbonate/phosphate ratio, most likely associated with the loss of labile carbonate, during cortical bone development the carbonate/phosphate ratio measured by Raman spectroscopy increases with time from embryos to adult [35] and in calvarial bones from postnatal days 3 to 6 months of age [39]. Fracture healing therefore appears as a recapitulation of the age dependent increase in carbonate content, with a leveling off of carbonate substitution as the bone matures. It is also possible that the cartilage areas examined contained less mineral, or some nonmineralized areas, phosphate contribution being weaker. This is not the case in intramembranous, woven, or control cortical bone, where mineral was present throughout the tissue and temporal changes were noted.

Previous studies suggested that crystallinity and collagen cross-linking correlated positively with bone strength [10, 40]. In this context, collagen maturity as measured by FTIRI was shown as related to the ratio of nonreducible pyridinoline to reducible deH-DHLNL collagen cross-links in connective tissues [41], and mineral maturity/crystallinity was related to the crystal size and perfection in the apatite *c*-axis direction as determined by X-ray diffraction [27]. In the healing fracture callus, collagen maturity only showed significant temporal increases in intramembranous bones. This suggests that in intramembranous bone, where mineralization proceeds directly on a type I collagen matrix throughout healing, maturation of the collagen along with the mineralization of that matrix can be detected by FTIRI. In the other tissues, results may be compromised by the presence of type X or type II and IX collagens which are present in calcified cartilage and are not stabilized by the same type of cross-links [42].

Acid phosphate substitution decreased continuously, with time, in several callus tissue components. This reflects the time dependent maturation of the hydroxyapatite crystal in which acid phosphate substitution is replaced by phosphate ions. In terms of acid phosphate substitution, it is important to note that although it was suggested previously that the ratio of 1128 cm^−1^/1096 cm^−1^ be used as the marker for acid phosphate substitution [28], that paper also indicated that the 1112 cm^−1^/1096 cm^−1^ ratio gave parallel results. In the newly formed tissues examined here, second derivative spectra, as well as visual examination of FTIRI spectra revealed a strong subband at 1112 cm^−1^. The ratio used here, therefore, was 1112 cm^−1^/1096 cm^−1^.

This study, demonstrating the usefulness of histologically resolved FTIRI data, does have limitations. First, fracture healing was only studied at one age and in one sex in the mouse, yet the healing process, at least in the mouse, is known to be sex- and age-specific [43, 44]. None the less, these data demonstrate the usefulness of FTIRI for future studies of drug effects in bone healing in the rodent fracture-model. Secondly, we relied on peak-height-ratios rather than curve fitting due to the number of spectra involved in the image, which of necessity, reduced the sensitivity of the assays. Finally, there was the possibility that nonmineralized areas in the images from calcified cartilage and endochondral bone might have been included. This, as noted above, would have artificially inflated the carbonate/phosphate ratio and decreased the mineral/matrix ratio in the two cartilage containing areas. The overall trends in those areas agreed with prior results, so we suspect this limitation is of less concern.

## 5. Conclusions

In conclusion, FTIRI can provide reproducible compositional data on the process of fracture healing in distinct histological zones. A series of validated parameters can be used to detail the composition and matrix qualities within the callus and newly formed bone. FTIRI will be useful for analysis of therapies for fracture healing in both preclinical and clinical models.

## Figures and Tables

**Table 1:** Stages of fracture healing in areas of endochondral bone formation as related to the different time points investigated by FT-IRI in the mouse. Intramembranous bone formation directly results in woven bone and hard callus without prior cartilage formation (stage 1 is excluded since this stage the fracture callus is an amorphous hematoma).

	Stage 1 inflammation	Stage 2 soft callus	Stage 3 hard callus	Stage 4 remodeling
		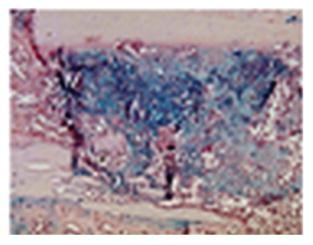	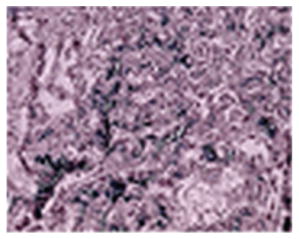	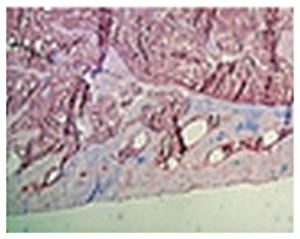
Time	Before week 1	Weeks 1 and 2	Week 2 and 4	Weeks 4 and 8
Predominant cell type	Inflammatory cells, platelets, macrophages	Chondrocytes, fibroblast, mesenchymal progenitors	Osteoblasts, “chondroclasts”	Osteoclast, osteoblast
Matrix	Hematoma, granulation tissue	ECM proteins (collagen II, Collagen X)	mineralized bone matrix, collagen I, woven bone	Lamellar bone, cortical and trabecular structure
Fracture healing processes	Reorganization migration of MSCs	Endochondral ossification, matrix mineralization	Vascular invasion, replacement of cartilage by bone	Bone and matrix degradation, new bone formation

**Table 2:** FT-IRI parameters described in the present study; HA = hydroxyapatite [7, 27, 28].

Parameter	Origin	Spectral measurement
Mineral/matrix	PO_4_^3−^ *ν*_1_ *ν*_3_/amide I	(916–1180 cm^−1^)/(1588–1712 cm^−1^) area ratio
Carbonate/phosphate	CO_3_^2−^ *ν*_3_/PO_4_^3−^ *ν*_1_ *ν*_3_	(852–890 cm^−1^)/(916–1180 cm^−1^) area ratio
HA crystallinity/maturity	Shape changes in PO_4_^3−^ *ν*_1_ *ν*_3_ mode	1030 cm^−1^/1020 cm^−1^ peak-height ratio
Collagen cross-links/maturity	Changes in amide I contour	1660 cm^−1^/1690 cm^−1^ peak-height ratio Valid for collagen types I and III
Acid phosphate content	Shape changes in PO_4_^3−^ *ν*_1_ *ν*_3_ mode Freshly precipitated, poorly crystalline HA/PO_4_^3−^ *ν*_3_	1112 cm^−1^/1096 cm^−1^ peak-height ratio

**Table 3:** Microcomputed tomography summary statistics.

Parameter	BV/TV *P* value	TMD *P* value	Callus volume *P* value
Week 1 versus 2	<**0.001**	0.172	**0.005**
Week 1 versus 4	**0.003**	**0.001**	0.068
Week 1 versus 8	**0.019**	**0.001**	0.320
Week 2 versus 4	0.189	0.051	0.677
Week 2 versus 8	0.051	**0.033**	0.254
Week 4 versus 8	0.881	0.990	0.853

Statistically sigificant values are shown in bold.

**Table 4:** Point-by-point comparisons of FTIRI Data (illustrated in Figure 5).

(a) Mineral/Matrix					

Week	Tissue	CO	EC	IM	WB
1	CC	<**0.001**	0.197	0.099	<**0.001**
	CO		<**0.001**	<**0.001**	<**0.001**
	EC			0.999	0.484
	IM				0.282

2	CC	**0.009**	0.966	0.999	0.998
	CO		<**0.001**	<**0.001**	**0.001**
	EC			0.421	0.121
	IM				0.999

4	CC	—	—	—	—
	CO		—	<**0.001**	**0.005**
	EC			—	—
	IM				0.542

8	CC	—	—	—	—
	CO		—	**0.020**	<**0.001**
	EC			—	—
	IM				0.971

Tissue	Week	2	4	8	

CC	1	<**0.001**	—	—	
	2		—	—	
	4			—	

CO	1	0.887	0.903	0.999	
	2		0.999	0.793	
	4			0.783	

EC	1	**0.009**	—	—	
	2		—	—	
	4			—	

IM	1	<**0.001**	<**0.001**	<**0.001**	
	2		0.160	0.340	
	4			0.999	

WB	1	**0.001**	<**0.001**	<**0.001**	
	2		0.628	**0.044**	
	4			0.946	

(b) Carbonate/phosphate					

Week	Tissue	CO	EC	IM	WB
1	CC	<**0.001**	0.579	0.982	<**0.001**
	CO		<**0.001**	**0.035**	0.082
	EC			0.933	0.357
	IM				0.324

2	CC	<**0.001**	<**0.001**	<**0.001**	<**0.001**
	CO		**0.028**	0.997	0.009
	EC			**0.048**	0.548
	IM				**0.003**

4	CC	—	—	—	—
	CO		—	0.826	0.999
	EC			—	—
	IM				0.939

8	CC	—	—	—	—
	CO		—	0.698	**0.003**
	EC			—	—
	IM				0.464

Tissue	Week	2	4	8	

CC	1	0.062	—	—	
	2		—	—	
	4			—	

CO	1	0.587	0.963	0.561	
	2		0.312	0.998	
	4			0.245	

EC	1	0.310	—	—	
	2		—	—	
	4			—	

IM	1	**0.012**	0.056	**0.009**	
	2		0.308	0.986	
	4			0.141	

WB	1	0.522	0.221	0.088	
	2		0.782	0.251	
	4			0.621	

(c) Crystallinity					

Week	Tissue	CO	EC	IM	WB
1	CC	**0.003**	**0.012**	**0.013**	<**0.001**
	CO		0.999	0.993	<**0.001**
	EC			0.998	<**0.001**
	IM				0.690

2	CC	**0.040**	0.124	**0.017**	0.084
	CO		0.987	0.084	0.744
	EC			0.273	0.999
	IM				<**0.001**

4	CC	—	—	—	—
	CO		—	0.659	0.641
	EC			—	—
	IM				0.979

8	CC	—	—	—	—
	CO		—	**0.468**	<**0.001**
	EC			—	—
	IM				**0.002**

Tissue	Week	2	4	8	

CC	1	0.337	—	—	
	2		—	—	
	4			—	

CO	1	0.827	0.999	0.999	
	2		0.917	0.870	
	4			0.999	

EC	1	0.909	—	—	
	2		—	—	
	4			—	

IM	1	0.417	0.999	0.985	
	2		0.456	0.603	
	4			0.995	

WB	1	<**0.001**	0.878	**0.013**	
	2		0.842	<**0.001**	
	4			0.129	

(d) Acid phosphate substitution					

Week	Tissue	CO	EC	IM	WB
1	CC	<**0.001**	**0.005**	<**0.001**	<**0.001**
	CO		<**0.001**	<**0.001**	<**0.001**
	EC			0.363	0.653
	IM				0.756

2	CC	<**0.001**	0.488	<**0.001**	**0.008**
	CO		<**0.001**	<**0.001**	<**0.001**
	EC			<**0.001**	**0.016**
	IM				0.534

4	CC	—	—	—	—
	CO		—	0.079	<**0.001**
	EC			—	—
	IM				0.254

8	CC	—	—	—	—
	CO		—	0.417	<**0.001**
	EC			—	—
	IM				**0.015**

Tissue	Week	2	4	8	

CC	1	0.680	—	—	
	2		—	—	
	4			—	

CO	1	0.998	0.998	0.847	
	2		0.999	0.892	
	4			0.870	

EC	1	0.645	—	—	
	2		—	—	
	4			—	

IM	1	<**0.001**	<**0.001**	<**0.001**	
	2		0.066	**0.035**	
	4			0.984	

WB	1	**0.002**	<**0.001**	<**0.001**	
	2		**0.041**	**0.017**	
	4			0.991	

(e) Collagen maturity/cross-link ratio					

Week	Tissue	CO	EC	IM	WB
1	CC	0.120	0.419	0.972	**0.005**
	CO		0.495	0.094	0.999
	EC			0.663	0.923
	IM				**0.019**

2	CC	0.471	0.250	0.494	0.799
	CO		0.119	0.999	0.918
	EC			0.089	0.156
	IM				0.891

4	CC	—	—	—	—
	CO		—	0.968	0.958
	EC			—	—
	IM				0.958

8	CC	—	—	—	—
	CO		—	**0.038**	0.193
	EC			—	—
	IM				0.823

Tissue	Week	2	4	8	

CC	1	0.855	—	—	
	2		—	—	
	4			—	

CO	1	0.999	0.256	0.723	
	2		0.234	0.831	
	4			**0.024**	

EC	1	0.060	—	—	
	2		—	—	
	4			—	

IM	1	**0.020**	<**0.001**	0.561	
	2		0.203	0.445	
	4			**0.008**	

WB	1	0.879	0.276	0.665	
	2		0.092	0.980	
	4			**0.044**	

Statistically significant *P* values are shown in bold. CO= cortical bone; CC = calcified cartilage; EC = endochondral bone; IM = intramembranous bone; WB = woven bone. Times are weeks after fracture.

## References

[1] Aspenberg P (2013). Special review: accelerating fracture repair in humans: a reading of old experiments and recent clinical trials. BoneKEy Reports.

[2] Tägil M, McDonald MM, Morse A (2010). Intermittent PTH(1–34) does not increase union rates in open rat femoral fractures and exhibits attenuated anabolic effects compared to closed fractures. Bone.

[3] Einhorn TA (2010). Can an anti-fracture agent heal fractures?. Clinical Cases in Mineral and Bone Metabolism.

[4] Roschger P, Paschalis EP, Fratzl P, Klaushofer K (2008). Bone mineralization density distribution in health and disease. Bone.

[5] Schindeler A, McDonald MM, Bokko P, Little DG (2008). Bone remodeling during fracture repair: the cellular picture. Seminars in Cell and Developmental Biology.

[6] Einhorn TA (1998). The cell and molecular biology of fracture healing. Clinical Orthopaedics and Related Research.

[7] Boskey AL, Di Masi E, Gower LB (2014). Infrared spectra and imaging. Biomineralization Sourcebook: Characterization of Biominerals and Biomimetic Materials.

[8] Gregory JS, Stewart A, Undrill PE, Reid DM, Aspden RM (2005). Bone shape, structure, and density as determinants of osteoporotic hip fracture: a pilot study investigating the combination of risk factors. Investigative Radiology.

[9] Gourion-Arsiquaud S, Allen MR, Burr DB, Vashishth D, Tang SY, Boskey AL (2010). Bisphosphonate treatment modifies canine bone mineral and matrix properties and their heterogeneity. Bone.

[10] Gourion-Arsiquaud S, Faibish D, Myers E (2009). Use of FTIR spectroscopic imaging to identify parameters associated with fragility fracture. Journal of Bone and Mineral Research.

[11] Willett TL, Pasquale J, Grynpas MD (2014). Collagen modifications in postmenopausal osteoporosis: advanced glycation end products may affect bone volume, structure and quality. Current Osteoporosis Reports.

[12] Camacho NP, Carroll P, Raggio CL (2003). Fourier transform infrared imaging spectroscopy (FT-IRIS) of mineralization in bisphosphonate-treated oim/oim mice. Calcified Tissue International.

[13] Gamsjaeger S, Buchinger B, Zoehrer R, Phipps R, Klaushofer K, Paschalis EP (2011). Effects of one year daily teriparatide treatment on trabecular bone material properties in postmenopausal osteoporotic women previously treated with alendronate or risedronate. Bone.

[14] Durchschlag E, Paschalis EP, Zoehrer R (2006). Bone material properties in trabecular bone from human iliac crest biopsies after 3- and 5-year treatment with risedronate. Journal of Bone and Mineral Research.

[15] Zoehrer R, Dempster DW, Bilezikian JP (2008). Bone quality determined by fourier transform infrared imaging analysis in mild primary hyperparathyroidism. The Journal of Clinical Endocrinology & Metabolism.

[16] Boskey AL, Lukashova L, Spevak L, Ma Y, Khan SR (2013). The kidney sodium-phosphate co-transporter alters bone quality in an age and gender specific manner. Bone.

[17] Paschalis EP, Shane E, Lyritis G, Skarantavos G, Mendelsohn R, Boskey AL (2004). Bone fragility and collagen cross-links. Journal of Bone and Mineral Research.

[18] Yang X, Ricciardi BF, Hernandez-Soria A, Shi Y, Pleshko Camacho N, Bostrom MPG (2007). Callus mineralization and maturation are delayed during fracture healing in interleukin-6 knockout mice. Bone.

[19] Kim M, Bi X, Horton WE, Spencer RG, Camacho NP (2005). Fourier transform infrared imaging spectroscopic analysis of tissue engineered cartilage: histologic and biochemical correlations. Journal of Biomedical Optics.

[20] Xia Y, Ramakrishnan N, Bidthanapally A (2007). The depth-dependent anisotropy of articular cartilage by Fourier-transform infrared imaging (FTIRI). Osteoarthritis and Cartilage.

[21] West PA, Bostrom MPG, Torzilli PA, Camacho NP (2004). Fourier transform infrared spectral analysis of degenerative cartilage: an infrared fiber optic probe and imaging study. Applied Spectroscopy.

[22] Bi X, Li G, Doty SB, Camacho NP (2005). A novel method for determination of collagen orientation in cartilage by Fourier transform infrared imaging spectroscopy (FT-IRIS). Osteoarthritis and Cartilage.

[23] Ouyang H, Sherman PJ, Paschalis EP, Boskey AL, Mendelsohn R (2004). Fourier transform infrared microscopic imaging: effects of estrogen and estrogen deficiency on fracture healing in rat femurs. Applied Spectroscopy.

[24] Voide R, van Lenthe GH, Müller R (2008). Differential effects of bone structural and material properties on bone competence in C57BL/6 and C3H/He inbred strains of mice. Calcified Tissue International.

[25] Beamer WG, Donahue LR, Rosen CJ, Baylink DJ (1996). Genetic variability in adult bone density among inbred strains of mice. Bone.

[26] Gardner MJ, van der Meulen MCH, Demetrakopoulos D, Wright TM, Myers ER, Bostrom MP (2006). In vivo cyclic axial compression affects bone healing in the mouse tibia. Journal of Orthopaedic Research.

[27] Pleshko N, Boskey A, Mendelsohn R (1991). Novel infrared spectroscopic method for the determination of crystallinity of hydroxyapatite minerals. Biophysical Journal.

[28] Spevak L, Flach CR, Hunter T, Mendelsohn R, Boskey A (2013). Fourier transform infrared spectroscopic imaging parameters describing acid phosphate substitution in biologic hydroxyapatite. Calcified Tissue International.

[29] Meganck JA, Begun DL, McElderry JD (2013). Fracture healing with alendronate treatment in the Brtl/+ mouse model of osteogenesis imperfecta. Bone.

[30] Turunen MJ, Lages S, Labrador A (2014). Evaluation of composition and mineral structure of callus tissue in rat femoral fracture. Journal of Biomedical Optics.

[31] Faibish D, Gomes A, Boivin G, Binderman I, Boskey A (2005). Infrared imaging of calcified tissue in bone biopsies from adults with osteomalacia. Bone.

[32] Verdelis K, Ling Y, Sreenath T (2008). DSPP effects on *in vivo* bone mineralization. Bone.

[33] Chiou HJ, Hung SC, Lin SY, Wu TK, Huang YT (2011). FT-IR microscopic imaging of calcified deposit of rotator cuff tendonitis: a pilot study and a randomised identification of the compositional components after extrusion from tendon to muscle. Vibrational Spectroscopy.

[34] Chiou HJ, Hung SC, Lin SY, Wei YS, Li MJ (2009). Correlations among mineral components, progressive calcification process and clinical symptoms of calcific tendonitis. Rheumatology.

[35] Raghavan M, Sahar ND, Kohn DH, Morris MD (2012). Age-specific profiles of tissue-level composition and mechanical properties in murine cortical bone. Bone.

[36] Fleet ME (2015). The carbonate ions in hydroxyapatite and biological apatite. Continuous Process Dynamics, Stability, Control and Automation.

[37] LeGeros RZ, Myers Karger H (1991). Calcium phosphates in oral biology and medicine. Monographs in Oral Sciences.

[38] Rey C, Shimizu M, Collins B, Glimcher MJ (1990). Resolution-enhanced Fourier transform infrared spectroscopy study of the environment of phosphate ions in the early deposits of a solid phase of calcium-phosphate in bone and enamel, and their evolution with age. I: investigations in the *v*_4_ PO_4_ domain. Calcified Tissue International.

[39] Tarnowski CP, Ignelzi MA, Morris MD (2002). Mineralization of developing mouse calvaria as revealed by raman microspectroscopy. Journal of Bone and Mineral Research.

[40] Yerramshetty JS, Akkus O (2008). The associations between mineral crystallinity and the mechanical properties of human cortical bone. Bone.

[41] Paschalis EP, Tatakis DN, Robins S (2011). Lathyrism-induced alterations in collagen cross-links influence the mechanical properties of bone material without affecting the mineral. Bone.

[42] Eyre DR, Dickson IR, Van Ness K (1988). Collagen cross-linking in human bone and articular cartilage. Age-related changes in the content of mature hydroxypyridinium residues. Biochemical Journal.

[43] Histing T, Stenger D, Kuntz S (2012). Increased osteoblast and osteoclast activity in female senescence-accelerated, osteoporotic SAMP6 mice during fracture healing. Journal of Surgical Research.

[44] Lopas LA, Belkin NS, Mutyaba PL, Gray CF, Hankenson KD, Ahn J (2014). Fractures in geriatric mice show decreased callus expansion and bone volume. Clinical Orthopaedics and Related Research.

